# Advancing the modified face name associative memory exam in cognitive aging research: insights into connectomic correlates and task reliability

**DOI:** 10.3389/fnagi.2025.1592678

**Published:** 2025-07-28

**Authors:** Yilin Liu, Mark H. Sundman, Dalen Hinderaker, Allison Yu-Chin Chen, Jacob M. Green, Lisbeth G. Haaheim, Hannah M. Siu, Catherine Jezerc, Kaitlyn Lai, Carol Chen, Parker Guss, Ying-hui Chou

**Affiliations:** ^1^Brain Imaging and TMS Laboratory, University of Arizona, Tucson, AZ, United States; ^2^Department of Psychology, University of Arizona, Tucson, AZ, United States; ^3^Evelyn F. McKnight Brain Institute, Arizona Center on Aging, and BIO5 Institute, University of Arizona, Tucson, AZ, United States

**Keywords:** associative memory, default mode network, medial temporal lobe, mild cognitive impairment, brain connectivity

## Abstract

**Introduction:**

The shift toward earlier detection in the Alzheimer's disease (AD) continuum underscores the need for more sensitive cognitive outcome assessments (COAs). Traditional COAs may lack precision in capturing cognitive dysfunction during preclinical stages. The Face-Name Associative Memory Exam (FNAME), a cross-modal task that integrates verbal and non-verbal memory, offers enhanced sensitivity and has shown associations with amyloid-β burden across the AD continuum, even in asymptomatic older adults.

**Methods:**

This manuscript reports on two experiments, broadening insights into this promising COA. Experiment 1 (descriptive observational, repeated-measures design) (*N* = 85) evaluates the alternate form reliability of a modified FNAME (mFNAME) by serially administering eight distinct versions of the task, revealing good reliability for mFNAME metrics and the absence of significant practice effects. Experiment 2 (cross-sectional observational design) (*N* = 32) examines structural and functional network topology to investigate neural correlates of mFNAME performance in non-demented older adults.

**Results:**

Experiment 1 demonstrated good alternate form reliability with no significant practice effect. Experiment 2 revealed significant associations between mFNAME performance and network properties like global efficiency, local efficiency, and system segregation in the default mode network (DMN) and medial temporal network (MTN). Subsequent analyses into more granular elements of the MTN and DMN revealed latent variables accounting for up to 44% of the covariance in mFNAME performance.

**Discussions:**

These findings deepen the understanding of the FNAME's psychometric properties and the neural correlates underlying task performance, providing insights into its utility as a sensitive COA early in the continuum of AD and related dementias.

## 1 Introduction

Amid the surge of secondary prevention trials targeting nascent stages of the Alzheimer's disease (AD) continuum, there is an imperative for neuropsychological assessments with enhanced sensitivity to quantify subtle cognitive changes in asymptomatic individuals. Traditional cognitive outcome assessments (COAs), designed to identify behavioral deficits associated with Mild Cognitive Impairment (MCI) or AD dementia, may not be sensitive enough to detect early changes that occur before clinical impairment becomes noticeable (Mortamais et al., 2017; Ritchie et al., 2017). Indeed, among cognitively normal (CN) older adults, studies utilizing conventional COAs commonly report a weak relationship between cognition and amyloid-β (Aβ) burden (Aizenstein et al., 2008; Jack et al., 2008; Storandt et al., 2009; Wirth et al., 2013). Furthermore, clinical trials for disease-modifying pharmacotherapies in the early stages of AD that use these conventional yet insensitive COAs as their primary or secondary outcome measures may experience ceiling effects, potentially underestimating treatment efficacy (Jutten et al., 2023). Thus, there is a need for COAs that are sensitive to cognitive deviations that may accompany clinically “silent” pathology affecting the medial temporal lobe (MTL) in the incipient stages of AD (Mortamais et al., 2017; Ritchie et al., 2017). One domain particularly well-suited for capturing these early deviations is associative memory.

Associative memory, the ability to link disparate pieces of information, is an essential cognitive process with heightened vulnerability to early AD pathology, particularly when binding cross-modal attributes (e.g., verbal and visual) (Chalfonte and Johnson, 1996; Goldstein et al., 2019; Naveh-Benjamin et al., 2004; Sperling et al., 2003; Stark and Squire, 2001; Werheid and Clare, 2007). Central to this cognitive domain, one COA with significant utility for discerning subtle deviations in cognitive aging is the Face-Name Associative Memory Exam (FNAME) (Rentz et al., 2011; Rubiño and Andrés, 2018). This cross-modal paired associative learning task evaluates an individual's ability to learn numerous unique face–name pairs. Since face–name binding is a ubiquitous demand of daily life, the FNAME has the advantage of tapping into ecologically relevant cognitive faculties, which can be lacking in conventional COAs (e.g., word list learning) (Loewenstein et al., 2018). Though often achieved automatically, this cognitive ability becomes increasingly taxing and salient as we age.

Among older adults, difficulty with names is commonly reported as a chief concern when discussing age-related changes in cognition—both learning the names of new acquaintances and recalling the names of wellknown individuals (James et al., 2008; Weaver Cargin et al., 2008). Interestingly, FNAME findings consistently reveal that face–name binding poses a unique challenge for the aging brain, significantly more so than face–occupation pairings (Rentz et al., 2017; Sanabria et al., 2018). The distinction in difficulty for face–name pairs is exemplified by findings that the term “Baker” is more readily associated with a face when presented as an occupation rather than as a name (McWeeny et al., 1987; Rubiño and Andrés, 2018; Young et al., 1986). This phenomenon underscores a unique feature of FNAME tasks; beyond cross-modal associative memory binding, it further taxes cognitive ability by requiring connections across abstract and unique dimensions that bypass semantic knowledge (Alviarez-Schulze et al., 2022). In addition to its inherent ecological validity, the FNAME has other favorable psychometric properties, such as the relative absence of ceiling effects (Rubiño and Andrés, 2018). This is a desirable feature for any COA intended to capture deviations among individuals with intact cognition and reduces the risk of Type 1 Error inflation that commonly confounds other AD COAs (Austin and Brunner, 2003; Carlson et al., 2022; Kueper et al., 2018). Another beneficial psychometric property of FNAME tasks is its cross-cultural validity, which is reflected in the emergence of versions for Spanish-, Czech-, Dutch-, and Greek-speaking populations (Alegret et al., 2020; Enriquez-Geppert et al., 2021; Kormas et al., 2020; Mazancova et al., 2017; Rubino et al., 2025; Vila-Castelar et al., 2020). In both Spain and Mexico, for example, the Spanish-FNAME has been demonstrated to be sensitive to age-related memory deficits (Flores Vazquez et al., 2021).

In AD research, findings consistently reveal a significant correlation between FNAME performance and Aβ burden, providing a valuable gradation of impairment across the disease continuum (Alegret et al., 2020; Rentz et al., 2010). Moreover, unlike conventional COAs, FNAME scores correspond with AD biomarker burden in asymptomatic CN cohorts in most (Fernandez-Alvarez et al., 2023; Rentz et al., 2011, 2010; Sanabria et al., 2018; Sperling et al., 2009; Vannini et al., 2012; Vila-Castelar et al., 2020; Young et al., 2023) but not all studies (Rentz et al., 2023). Functional neuroimaging during the FNAME task further reveals aberrant task-related neural activation patterns in Aβ-positive CN older adults (Sperling et al., 2009; Vannini et al., 2012). Critically, this amyloid-dependent network dysfunction observed during FNAME tasks in preclinical AD mirrors findings from patients with MCI and AD (Celone et al., 2006; Pihlajamaki et al., 2008). Further highlighting its suitability for secondary prevention trials, a comprehensive meta-analysis of longitudinal studies identified the FNAME as one of the most robust predictive tools for assessing AD progression risk (Belleville et al., 2017). Among 61 evaluated COAs, the FNAME emerged as the top performer, boasting 100% sensitivity and ranking as one of only five COAs with a sensitivity above 90% (Belleville et al., 2017). There are, however, several gaps in the FNAME literature that we will address in this manuscript with two distinct experiments.

**Experiment 1** examines the reliability of a modified FNAME (mFNAME, described in Section 2.2) as a serially administered COA in longitudinal studies. While it is essential to document cognitive decline longitudinally in AD research, practice effects frequently complicate the interpretation of repeated COAs and obscure the actual progression of cognition over time. Practice effects can be categorized as either generalized task familiarity (i.e., strategy development and reduced test anxiety) or the specific recollection of test items from previous encounters, termed “learning over repeated exposures” (LORE). Utilizing alternate versions is known to mitigate LORE, but lingering generalized practice effects may remain (Beglinger et al., 2005; Benedict and Zgaljardic, 1998). This manuscript evaluates the alternate form reliability of mFNAME performance and practice effects by serially administering eight distinct versions of the task.

**Experiment 2** examines functional and structural connectivity profiles to elucidate the neural correlates of mFNAME task performance in older adults. Previous functional neuroimaging studies illustrate that successfully forming and recalling face–name pairs necessitates synchronized activity across a distributed memory network (Miller et al., 2008; Sperling et al., 2003; Vannini et al., 2011; Zeineh et al., 2003). This network includes the hippocampus and neighboring medial temporal structures but also encompasses a broad array of cortical areas, such as the precuneus and posterior cingulate, highlighting the complexity and distributed nature of associative memory processing (Miller et al., 2008; Vannini et al., 2011). While these distributed task activation patterns are well elucidated, less is understood about how task performance corresponds with structural and functional network topology. Structural and functional connectomes are highly variable with age and are known to influence cognitive processes (Andrews-Hanna et al., 2007; Chen et al., 2009), but, to our knowledge, no cognitive aging studies have reported the relationship between network topology and face–name associative learning.

Thus, the objective of **Experiment 2** is to elucidate the relationships between various brain network properties and mFNAME performance in cognitively impaired older adults without dementia. To fulfill this objective, we utilized graph theoretical analyses to derive properties of the brain's connectome, such as global efficiency, local efficiency, and system segregation, which may be more sensitive to age-related changes in network organization (Chan et al., 2014; Ewers et al., 2021; Sala-Llonch et al., 2014). Similar network analyses have also demonstrated efficacy in revealing atypical network reorganization along the AD continuum, with consistent reports of reduced network efficiency (Berlot et al., 2016; Reijmer et al., 2013; Shu et al., 2012; Wright et al., 2021). These findings underscore the utility of graph-based analyses in identifying critical network disruptions underlying age and AD-related cognitive decline. In this study, we extend these approaches to both resting-state functional and structural connectivity to delineate how alterations in cortical network topology—particularly changes in hub efficiency and system integration—are associated with performance on the mFNAME task.

The overarching goal of this study is to evaluate both the psychometric properties and the neurobiological relevance of the modified Face-Name Associative Memory Exam (mFNAME) as a candidate cognitive outcome assessment for early-stage AD research. We hypothesize that (1) mFNAME performance will demonstrate strong alternate form reliability with minimal practice effects (Experiment 1), and (2) task performance will be associated with connectivity metrics in brain networks implicated in memory and aging among individuals with MCI. By combining behavioral reliability analysis with graph theoretical metrics of brain structure and function, this study aims to provide foundational support for the use of mFNAME as a reliable, sensitive, and neurobiologically informed tool for cognitive aging research.

## 2 Methods

### 2.1 Experimental design

Experiments 1 and 2 comprised distinct cohorts with no overlap in participation.

**Experiment 1** (Alternate Form Reliability and Practice Effects) initially enrolled 152 young adults from the University of Arizona. Of those, 85 young adults completed all eight versions of the mFNAME (mean age = 21.9 years; 57 females). This study followed a descriptive observational repeated-measures design, in which young adult participants completed all eight versions of the mFNAME to assess reliability and practice effects. Participants were recruited using non-probability sampling through the university subject pool. The primary outcome measures were overall task accuracy and the sensitivity index (D1), both of which reflect core aspects of associative memory performance. Hit rate and false alarm rate were examined as secondary outcome measures to further characterize response patterns. Additional details are provided in Section 2.2. Experiment 1 was conducted with young adults who were encouraged to participate in research studies for educational purposes, leveraging the enhanced feasibility that this population offers in a university setting.

**Experiment 2** (Network Topology) enrolled 32 older adults with MCI from the Tucson area community (mean age = 65.8 ± 7.43 years; 27 females; mean education = 16.36 ± 2.19 years). This study employed a cross-sectional observational design to examine associations between structural and functional network topology and mFNAME performance in individuals with MCI. Participants were recruited using non-probability sampling through community outreach and local advertisements. Primary outcome measures included mFNAME performance scores and graph theory metrics (e.g., global efficiency, local efficiency, and system segregation) derived from structural and functional connectivity data. Additional details are provided in Section 2.6. MCI diagnosis was established using the revised Mayo Clinic criteria, which included self- or informant-reported cognitive complaints, objective cognitive impairment, preserved independence in daily functioning, and the absence of dementia. The MCI diagnosis was supported by the National Alzheimer's Coordinating Center Uniform Data Set Neuropsychological Battery, Version 3 (UDSNB-3) (Weintraub et al., 2018). For each participant, Z-scores were calculated and adjusted for age, sex, and educational level. These normalized scores were then used to classify participants as MCI, following the ‘comprehensive criteria' outlined by Bondi et al. (2014).

The University Institutional Review Board reviewed and approved all protocol procedures. Exclusion criteria for both cohorts included (1) self-reported clinical history of brain injury, cardiovascular disease, or other neurological conditions such as dementia or Parkinson's disease, and (2) untreated psychiatric symptoms that meet DSM-IV criteria, including depression, anxiety, and substance use disorders. Specific to our cohort of older adults in Experiment 2, we assessed any contraindications for Magnetic Resonance Imaging (MRI) that might present safety concerns.

### 2.2 Modified face-name associative memory exam (mFNAME)

Psychopy software was employed to display stimuli and gather responses for the mFNAME, which consists of two phases: encoding and retrieval. During the encoding phase, participants were instructed to memorize the face–name pairs and make a subjective decision on how well the name fit the corresponding face on a scale from 1 (poor match) to 4 (good match), a task component designed to augment associative encoding (Carr et al., 2017). For the encoding phase, participants were presented with 24 face–name pairs. Following a 5-min break, during the retrieval phase, participants were instructed to decide whether the presented face–name pairs were “Correct” (i.e., the face–name pairs matched those presented during the encoding phase), “Incorrect” (i.e., the faces and names were presented during the encoding phase but in the wrong combination), or “New” (i.e., neither the names nor the faces were presented during the encoding phase). In total, there were 36 pairs consisting of 12 “Correct,” 12 “Incorrect,” and 12 “New” pairs during the retrieval phase, with each displayed pair presented for a duration of 5.5 s. Faces were adopted from the Chicago Face Database and were randomly and evenly distributed according to race, gender, and age into eight parallel versions. Table 1 is provided to highlight the comparability of the mFNAME task and the original FNAME introduced by Rentz et al. (2011).

**Table 1:** Comparison of the original FNAME and modified FNAME (mFNAME).

**Feature**	**mFNAME (modified version)**	**Original FNAME (Rentz et al., 2011)**
Face/Occupation Pairs	No	Yes (face–occupation pairs included)
Number of Pairs	24 face–name pairs (encoding); 36 total pairs in retrieval	32 total (16 face–name, 16 face–occupation)
Encoding Task	Rate face–name fit on a scale (1-4)	Study phase with examiner-led name association
Encoding Duration per Item	5.5 s per pair	2 s per face in study phase
Retrieval Task	Correct (original pair), Incorrect (mismatched pair), New (never seen before)	Face presented, participant recalls name
Retrieval Stimuli Count	12 Correct, 12 Incorrect, 12 New	16 face–name pairs, 16 face–occupation pairs
Response Options	Correct, Incorrect, New	Free recall and cued recall
Break Between Phases	5 min	Not specified
Face Source	Chicago face database	Not specified
Number of Versions	8 parallel versions	Not specified

In **Experiment 1**, participants serially completed all eight versions of the mFNAME task, which were administered in a randomized sequence to mitigate potential learning-order effects. The tasks were unsupervised and completed on their personal computers. Participants could complete only one mFNAME task per day, and the average time to complete all eight versions of the mFNAME was 17 ± 9 days.

In **Experiment 2**, a different cohort of participants completed a single mFNAME task selected randomly from the eight versions. Research staff supervised the completion of this task following the acquisition of multimodal MRI scans.

### 2.3 MRI acquisition (Experiment 2)

Before administering the mFNAME, MRI data were acquired using a MAGNETOM^®^ Skyra 3 Tesla MRI scanner (Siemens Medical Systems, Erlangen, Germany) with a 32-channel receiver head coil. Foam pads were applied to prevent head motion. The structural MRI protocol included T1-MPRAGE (a 3D gradient echo pulse sequence, T1-weighted) with TR = 2,530 ms, TE = 3.3 ms, TI = 1,100 ms, FA = 7°, FoV = 256 × 256 mm^2^, parallel imaging (GRAPPA 2), resolution: 1 × 1 × 1 mm^3^, and T2-FLAIR (a fluid-attenuated inversion recovery MRI sequence, T2-weighted) with TR = 6,700 ms, TE = 101 ms, TI = 2,500 ms, FA = 120°, FoV = 256 × 256 mm^2^, parallel imaging (GRAPPA 2), resolution: 1 × 1 × 2.5 mm^3^; scan time = 8 min. Diffusion-weighted MRI (single-shot parallel and multi-band dual-spin-echo EPI pulse sequence) parameters included FoV= 256 × 256 mm^2^; in-plane matrix size = 128 × 128; in-plane acceleration factor = 2; multi-band factor = 2; TE = 119 msec; TR = 3,700 msec; slice thickness = 2 mm; voxel size = 2 mm^3^; b = 0, 1,000, 2,000, and 3,000 s/mm^2^ as three shell acquisitions for further high angular resolution diffusion imaging (Dykes et al., 2009); number of diffusion-encoding directions = 60; scan time = 9 min. Finally, a resting-state fMRI (rs-fMRI) (T2^*^-weighted gradient-echo EPI pulse sequence, FoV = 240 × 240 mm^2^; TR = 3,000 msec; TE = 36 msec; flip angle = 90°; in-plane acquisition matrix size = 160 × 160; voxel size = 1.5 mm^3^; and multi-band factor = 2; scan time = 8 min) was acquired. During the scans, participants were asked to stay awake and hold still, keep their eyes focused on a displayed crosshair, and allow their thoughts to come and go freely.

### 2.4 MRI preprocessing (Experiment 2)

All MRI data were converted to the Brain Imaging Data Structure (BIDS) format using custom scripts. After BIDS conversion, preprocessing was performed on the T1-weighted and rs-fMRI data using fMRIPrep v1.0.3 (Esteban et al., 2019). This included slice time correction, motion alignment, and field distortion correction using a field map. Subsequently, co-registration to the corresponding T1-weighted space was executed using boundary-based registration. Each T1-weighted volume was further corrected for intensity, non-uniformity, and skull stripping.

To process the structural diffusion-weighted imaging (DWI) data, we used a custom shell script. For DTI preprocessing and whole-brain tractography, the following steps were implemented using MRtrix3 and FSL: (1) combining raw DWI, diffusion b-values, and vectors into one matrix; (2) denoising with *dwidenoise* (MRtrix3); (3) correcting Gibbs artifact and eddy currents using eddy (FSL); and (4) bias field correction (ANTs). Brain extraction on the B0 images was also conducted using *dwi2mask* (MRtrix3). The response function was estimated by *dwi2response*. Subsequently, fiber orientation distributions were evaluated using *dwi2fod* and the msmt_csd algorithm. The individual T1 brain image was segmented using FreeSurfer v7.1.1 (https://surfer-nmr-mgh-harvard-edu.ezproxy4.library.arizona.edu/) incorporating the Human Connectome Project's Multimodal Parcellation (HCP-MMP v1.0) atlas, which consists of 180 cortical (Glasser et al., 2016) and nine subcortical regions. Furthermore, FreeSurfer was applied to both T1 and T2 MRIs to obtain a more reliable segmentation of the hippocampal subfields with enhanced tissue contrast and landmarks of the internal hippocampal structure. This enabled the construction of a 379 × 379 whole-brain structural connectome matrix, including the brainstem for whole-brain tractography, which was used for graph-theoretical (network-based) analysis.

For the preprocessing of resting-state fMRI data with fMRIprep, physiological noise and other non-neuronal fluctuations were nuisance regressors were accounted for by incorporating, such as signals from cerebrospinal fluid (CSF), white matter (WM), and the global signal, along with their derivatives, quadratic terms, squares of derivatives, and head motion estimates, were incorporated in the first-level general linear regression model. To refine our analysis, the residuals from the nuisance regression underwent a bandpass filter between the frequencies of 0.01 and 0.1 Hz. Additionally, spatial smoothing of functional data was performed using a 5-mm full-width half-maximum (FWHM) Gaussian kernel. For each ROI and each participant, we first obtained the average time series for all the voxels in the ROI and then normalized (z-scored) the ROI time series. The normalized resting-state fMRI time series for each ROI was then correlated with the corresponding value for each remaining ROI, yielding a 379 × 379 correlation matrix for each participant. The Pearson r correlation value in each matrix cell was transformed to Fisher-z.

### 2.5 Network construction (Experiment 2)

Graph theory serves as a mathematical instrument for quantifying the topology of systems that can be modeled as networks. In this framework, a network comprises various entities, known as nodes, which are linked by a series of connections, known as edges. Here, the 379 ROIs were the network nodes, and the edges represented the structural or functional connections between them. Many studies have used previously defined sets of modules based on patterns of resting-state or task-related fMRI data (Power et al., 2011; Yeo et al., 2011). We adopted the network parcellation generated by Barnett and colleagues, which contains eight modules that closely align with the literature (Barnett et al., 2021). After defining the nodes in each network, brain networks were constructed with a specified sparsity. The sparsity threshold ensures that all resultant networks have comparable topological structures with the same number of edges (Wang et al., 2011). To generate a weighted undirected network, we applied a range of sparsity thresholds (0.2, 0.3, 0.4, and 0.5) suggested by the literature (Geng et al., 2017) and observed no impact of the sparsity threshold on the results. We utilized a sparsity threshold of 0.4 for the graph theoretical analysis described below.

### 2.6 Graph theoretical analysis (Experiment 2)

We used the Brain Connectivity Toolbox to calculate three graph theoretical measures: global efficiency, local efficiency, and system segregation (Rubinov and Sporns, 2010). Detailed descriptions of these metrics are given below:

Global efficiency is a measure that quantifies the efficiency of information transfer across an entire network/module. It is computed as the average inverse of the shortest path length between all pairs of nodes. The “shortest path length” refers to the minimum number of edges (or connections) that must be traversed to move from one node to another. By taking the inverse of these path lengths and averaging them, we obtain a higher global efficiency measure when paths are short (and thus efficient). A network with high global efficiency indicates that, on average, information can be quickly and efficiently transferred between nodes in the network. This measure provides insights into the brain's ability to integratively process information across distributed brain regions (Deery et al., 2023).

Local efficiency focuses on information transfer within each node's immediate vicinity or neighborhood. It is determined by calculating the average shortest path length of each node to the rest of the nodes within its module or network. High local efficiency indicates that even if a particular node were removed or malfunctioned, its immediate neighbors could still communicate efficiently. This measure provides insights into the network's resilience and fault tolerance at a localized level (Deery et al., 2023).

System segregation quantifies the extent of separation among network modules, determined by comparing the strength of connections within modules to those between different modules. For diffusion-weighted imaging (DWI) data, these strengths are measured by the mean number of streamlines connecting nodes. In the context of resting-state functional MRI (fMRI) data, they are defined by the mean normalized correlations. Higher values in both modularity and system segregation metrics indicate a more distinctly segregated network. System segregation is computed as an average at the whole-brain level, incorporating all nodes, while at the module level, it is determined based on the nodes within each specific module. This measure reflects the brain's ability to organize processing into specialized networks with reduced interference from unrelated information (Deery et al., 2023).

### 2.7 Statistical analysis

Four outcome measures were derived from the mFNAME. First, absolute accuracy was computed by tallying the total number of accurate responses across all trials. Second, following Carr et al. (2017), we categorized the recognition results into nine groups (3 × 3) based on three types of face–name pairs (i.e., Correct, Incorrect, and New pairs) and three types of responses (i.e., Correct, Incorrect, and New pairs). Additionally, the hit rate represented the rate of accurately responding “Correct” to “Correct” pairs (CC), while the false alarm rate was defined as the rate of wrongly responding “Correct” to “Incorrect” pairs (CI). Finally, a sensitivity index (D1) that quantified the ability to distinguish between signal and noise was defined as Z (hit rate) – Z (false alarm rate).

For **Experiment 1**, alternate form reliability for mFNAME was assessed using intraclass correlation coefficients (ICC) based on a two-way mixed-effects model, focusing on absolute agreement. The ICC quantifies the proportion of total variance attributed to between-subject differences, yielding a value between 0 and 1. The coefficients can be used to examine the consistency of observed values between samples and visits, facilitate comparisons between different test versions. Interpretation of ICC values follows established guidelines: poor (< 0.5), moderate (0.5 to 0.74), good (0.75 to 0.9), and excellent (>0.9) (Koo and Li, 2016).

Alongside the ICC, we also computed each metric's standard error of measurement (SEM). The SEM, an absolute reliability measure, quantifies error in the same units as the original measurement. It provides insights into the precision of individual scores, addressing individual variations that contribute to the ICC. Conceptually, the SEM indicates the expected fluctuations in an individual's score due to random errors across repeated test administrations. The SEM is determined using the formula: SEM = $\sqrt{SD^{2}\;*\left(1-ICC\right)}$. Lastly, we computed the Minimal Detectable Change (MDC), which denotes the smallest score change considered significant beyond mere measurement error (Donoghue et al., 2009). The MDC is instrumental in determining whether scores reflect a meaningful change over time that cannot be attributed to random variability. The MDC was estimated at the 95% confidence interval (CI) using the function MDC_95_ = z-value × SEM × √2.

Furthermore, to assess potential practice effects, we used a linear mixed-effects model to examine changes in FNAME performance over time (Laird and Ware, 1982). This approach allowed us to include all time points for each participant and minimize bias. The model included the main fixed effect for time (8 time points) and random intercepts to account for within-subject correlation.

For **Experiment 2**, eight structural and eight functional networks (i.e., default mode, medial temporal, frontal parietal, salient, visual, language, somatosensory, and auditory networks) were selected for subsequent analyses. We extracted global efficiency, local efficiency, and system segregation metrics for each network. To examine how network topology corresponds with mFNAME performance, a regression model evaluated these network properties alongside mFNAME accuracy and sensitivity (D1). This preliminary analysis enabled us to identify the network(s) with the strongest association with mFNAME measures.

Following our graph-theory enabled network selection, we conducted a behavioral partial least squares analysis (PLSC) to analyze the relationship between FNAME performance and more granular brain network properties. PLSC is a multivariate latent variable technique adept at handling collinear data and exploring relationships between different data modalities, such as our connectomic and behavioral measures (Abdi and Williams, 2013; Krishnan et al., 2011). It functions by decomposing highly correlated data matrices into singular vectors via singular value decomposition (SVD). For this PLSC, we compiled a data matrix with (X) comprising network properties and (Y) containing FNAME outcome measures for all participants. Subsequent matrix decomposition yielded latent variables (LVs), each representing the shared variance between connectomic properties (X) and behavioral outcome measures (Y). These LVs capture the underlying patterns linking brain connectivity and FNAME performance.

A two-step approach was employed to assess the statistical significance of the LVs and identify brain regions with significant contributions. First, the statistical significance of each LV was assessed with non-parametric permutation testing by re-running the PLSC on each permutation sample, creating a distribution to calculate *p-values* for each LV (Krishnan et al., 2011). LVs with *p-values* < 0.05 after 1,000 permutations were considered statistically significant (Abdi and Williams, 2013). Next, a bootstrap analysis was conducted to assess the contribution of different brain regions in the selected network(s). ROIs were evaluated based on a bootstrap ratio with an absolute value greater than or equal to 1.96 (corresponding to 95% CI), determined by 1,000 resampling's. This threshold identified ROIs in the LVs that significantly accounted for the covariance in the data.

The *p-values* for each LV were corrected using the Benjamini-Hochberg false discovery rate (FDR) method to account for multiple comparisons. We set the threshold for statistical significance at *P* < 0.05 after the false discovery rate correction.

## 3 Results

### 3.1 Experiment 1: alternate form reliability and practice effects of modified face-name associative memory exam (mFNAME)

**Figure 1:**
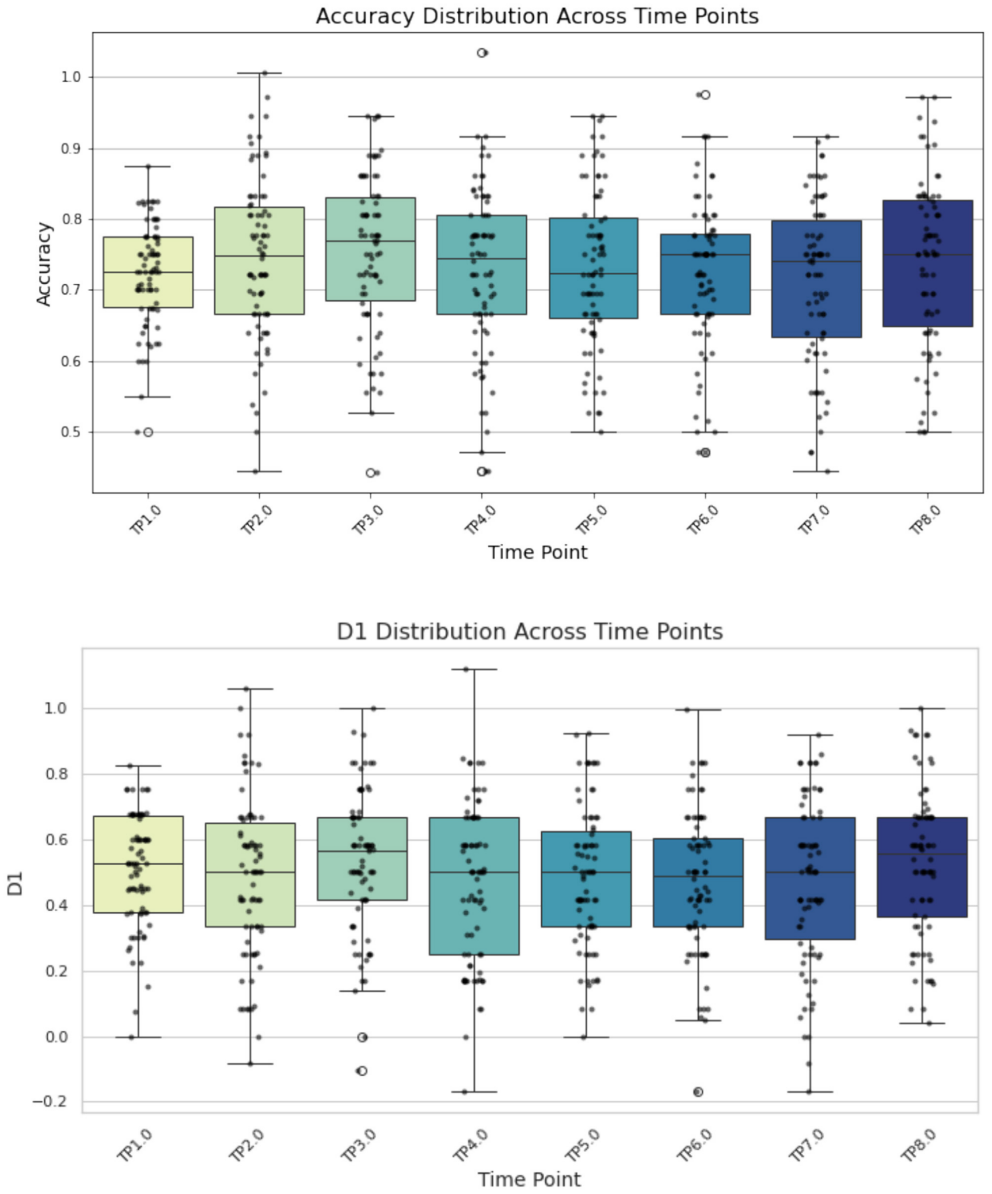
Results from the linear mixed effects model examining mFNAME primary outcome measures across all time points.

Of the 85 young adults who completed all eight versions of the mFNAME, seven individuals were identified as outliers due to overall reaction times that exceeded three standard deviations and were excluded from subsequent analyses. Table 2 summarizes the intraclass correlation coefficient (ICC), 95% confidence interval (CI), standard error of measurement (SEM), and minimal detectable change (MDC) values for all mFNAME metrics. All the ICC values were between 0.8 and 0.9, indicating good alternate form reliability. In our evaluation of practice effects, the linear mixed effects model indicated no significant changes in mFNAME D1, hit rate, false alarm rate, and accuracy scores across all time points after correction for multiple comparisons (Figure 1). Notably, although one of the *post-hoc* analyses revealed a significant improvement in accuracy at time point 3 compared to time point 1 (*p* = 0.0382), this result did not survive correction for multiple comparisons. Overall, the analysis suggests that, even when administered to healthy young adults in close succession, the mFNAME is resilient to generalized practice effects.

**Table 2:** Results of the alternate form reliability and the minimal detectable change of the mFNAME in healthy participants (*N* = 78).

**Metrics**	**ICC (3, k)**	**CI 95%**	**SEM**	**MDC_95_**
Accuracy	0.90	[0.87, 0.93]	0.041	0.114
D1	0.84	[0.77, 0.89]	0.098	0.272
Hit Rate	0.82	[0.74, 0.87]	0.073	0.201
False Alarm	0.80	[0.72, 0.86]	0.078	0.215

CI, confidence interval; ICC, intraclass correlation coefficient; MDC_95_, the minimal detectable change calculated based on 95% confidence interval; SEM, standard error of measurement.

### 3.2 Experiment 2: network topology and mFNAME performance in older adults

#### 3.2.1 Univariate relationship between structural connectivity and mFNAME performance

The regression model comprising behavioral outcome measures and graph theory properties across eight structural networks revealed significant associations between mFNAME variables and the default mode network (DMN) and the medial temporal network (MTN). Specifically, mFNAME performance was positively correlated with global efficiency and local efficiency for the structural DMN and MTN (Table 3). Additional analyses of mFNAME performance and its correlation with the properties of other structural networks are provided in the Supplementary Table 1.

**Table 3:** Outcomes of regression analyses examining the relationships between structural network properties (global efficiency, local efficiency, and system segregation) and mFNAME performance metrics, specifically accuracy and sensitivity index D1.

**Structural network**		**Accuracy**		**D1**	
		**R-value**	***p-value***	**R-value**	***p-value***
Global Efficiency	Medial temporal network	0.5416	0.0152^*^	0.5663	0.0239^*^
	Default mode network	0.6012	0.0075^**^	0.5059	0.0376^*^
Local Efficiency	Medial temporal network	0.4886	0.0239^*^	0.3421	0.0871
	Default mode network	0.5274	0.0108^*^	0.4756	0.0376^*^
System Segregation	Medial temporal network	0.2980	0.1672	0.3631	0.0884
	Default mode network	0.0609	0.7675	−0.0602	0.7699

The results are reported after applying FDR correction for multiple comparisons. Asterisks indicate levels of statistical significance: ^*^p < 0.05, ^**^p < 0.01, ^***^p < 0.001.

#### 3.2.2 Univariate relationship between functional connectivity and mFNAME performance

For resting-state functional connectivity, the MTN and DMN remained the most strongly associated with mFNAME task performance. We observed a significant positive association between the system segregation in the functional MTN with mFNAME accuracy and sensitivity index D1 (Table 4). Additionally, system segregation of the functional DMN is significantly associated with mFNAME accuracy. However, unlike the structural network properties reported above, global and local efficiency of the functional DMN and MTN are not significantly associated with FNAME performance. Additional analyses of the relationship between mFNAME and the properties of other functional networks are provided in the Supplementary Table 2.

**Table 4:** Outcomes of regression analyses examining the relationships between functional network properties (global efficiency, local efficiency, and system segregation) and mFNAME performance metrics, specifically accuracy and sensitivity index D1.

**Functional network**		**Accuracy**		**D1**	
		**R-value**	***p-value***	**R-value**	***p-value***
Global Efficiency	Medial temporal network	0.0322	0.8587	−0.1469	0.4145
	Default mode network	0.2124	0.4705	0.0525	0.7713
Local Efficiency	Medial temporal network	0.1910	0.29499	0.0184	0.9200
	Default mode network	0.1517	0.4071	−0.0306	0.8677
System Segregation	Medial temporal network	0.5644	0.0040^**^	0.5482	0.0055^**^
	Default mode network	0.4103	0.0464^*^	0.3434	0.1003

The results are reported after applying FDR correction for multiple comparisons. Asterisks indicate levels of statistical significance: ^*^p < 0.05, ^**^p < 0.01, ^***^p < 0.001.

#### 3.2.3 Topological features of the DNM and MTN associated with mFNAME performance

**Figure 2:**
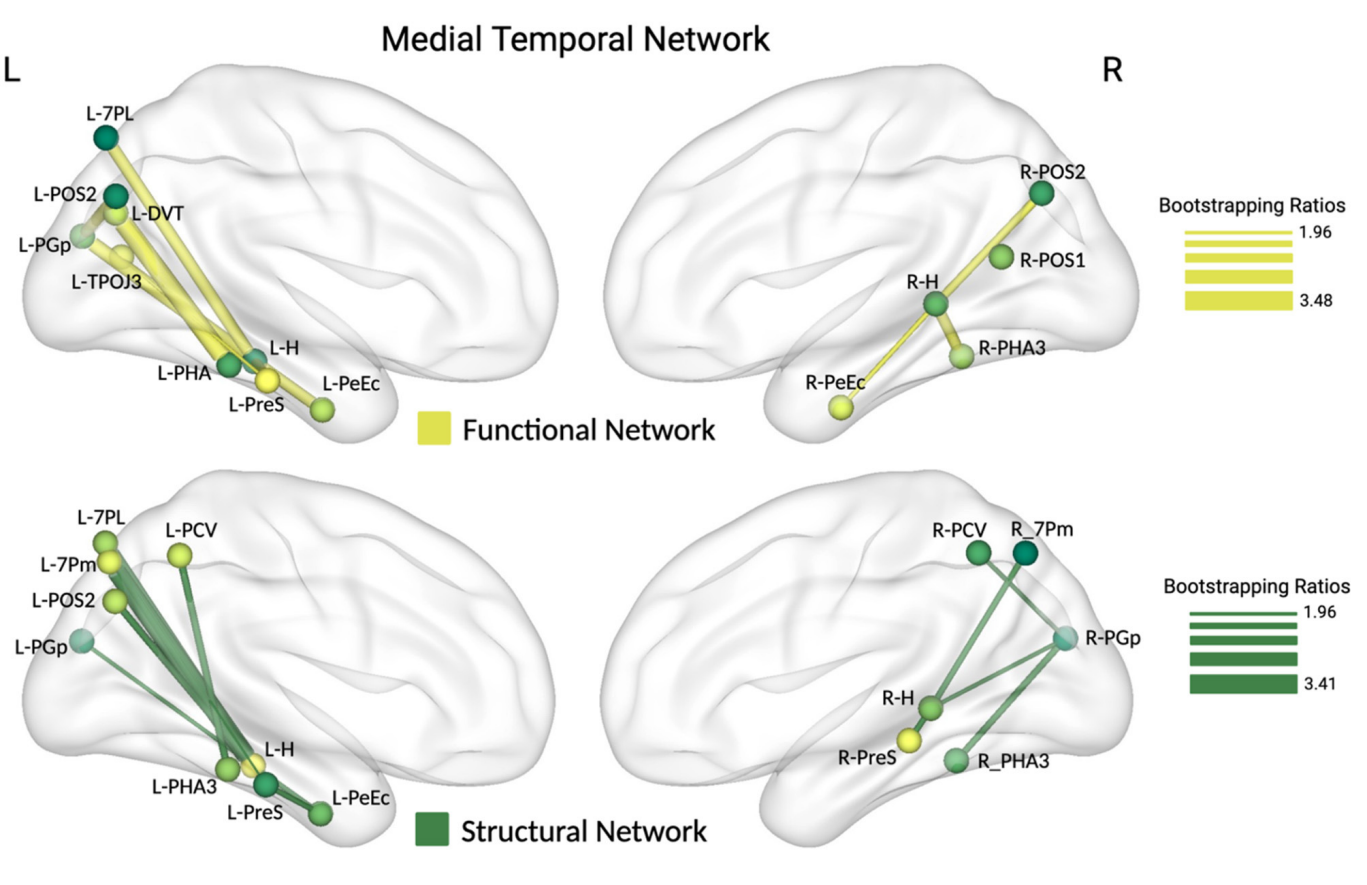
Partial least squares correlation (PLSC) analysis was conducted to examine the association between structural and functional connectivity within nodes of the medial temporal network (MTN) and mFNAME performance. The figure highlights within-MTN connectivity positively correlated with mFNAME performance, supported by a threshold bootstrap ratio of 1.96. Thicker lines represent higher bootstrap ratios, indicating a stronger association with mFNAME performance. The brain is viewed from the medial perspective.

To elucidate the contributions of individual MTN and DMN nodes to mFNAME performance, we applied partial least squares correlation (PLSC) analysis to examine the topological features. First, evaluating the resting-state functional connectivity of the MTN, we identified a significant latent variable accounting for 44% of the covariance. Bootstrapping analysis revealed that mFNAME performance is positively associated with functional connectivity between several brain regions within the functional MTN, including the superior parietal lobe (L_7PL), inferior parietal lobe (L_PGp), posterior cingulate cortex (L_DVT, L_POS2, R_POS2, R_POS1, L_TPOJ3), medial temporal lobe (L_PreS, L_PeEc, R_PeEc, L_Subi, R_Subi, L_TPOJ3, L_PHA3, R_PHA3), and hippocampal subfields (L_CA3, L_GC-DG, R_CA3, R_CA4, R_GC-DG) (Figure 2). Similarly, when examining the relationship between the structural connectivity of the MTN and FNAME performance, a significant latent variable emerged, explaining 31% of the covariance. Bootstrapping analysis indicated an association between higher FNAME scores and increased structural connectivity involving the superior parietal lobe (L_7PL, L_7Pm, R_7Pm), posterior cingulate cortex (L_POS2, L_PCV, R_PCV), medial temporal lobe (L_PreS, R_PreS, L_PHA3, L_PeEc, R_Subi), inferior parietal lobe (L_PGp, R_PGp), and hippocampal subfields (L_CA3, L_CA4, L_GC-DG, R_GC-DG, R_CA1, R_CA3). These findings reveal the connectomic features of the structural and functional MTN associated with performance on mFNAME tasks in older adults with cognitive impairment (Figure 2).

**Figure 3:**
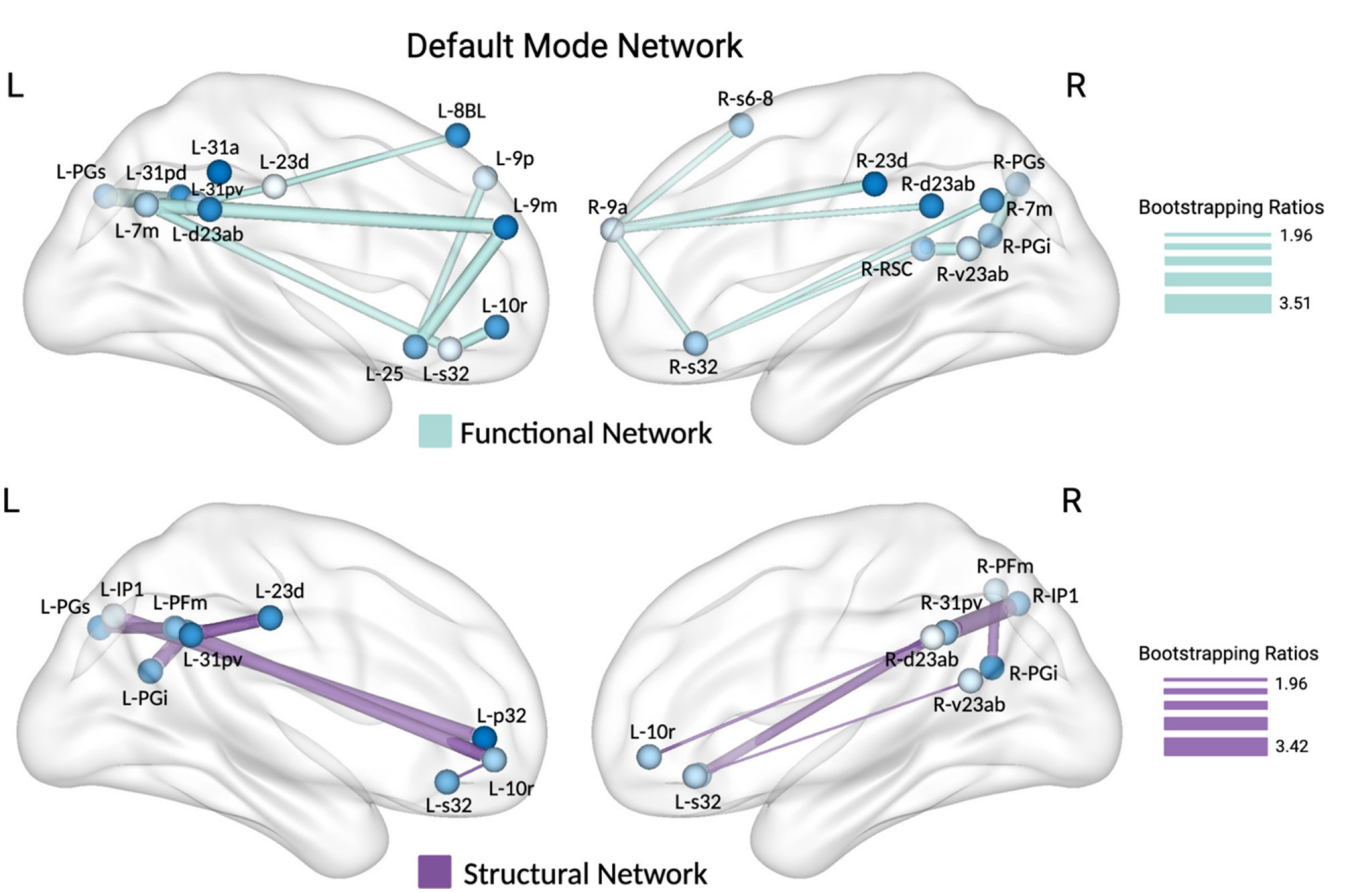
Partial least squares correlation (PLSC) analysis was conducted to examine the association between structural and functional connectivity within nodes of the default mode network (DMN) and FNAME performance. The figure highlights within-DMN connectivity positively correlated with mFNAME performance, supported by a threshold bootstrap ratio of 1.96. Thicker lines represent higher bootstrap ratios, indicating a stronger association with mFNAME performance. The brain is viewed from the medial perspective.

For the DMN, the analysis of functional network revealed a significant latent variable accounting for 37% of the variance, particularly within the medial prefrontal cortex (L_10r, L_9m, L_25, L_s32, R_s32, L_9m), dorsomedial prefrontal area (L_8BL, L_9p, R_9a, R_s6-8), posterior cingulate cortex (L_31a, L_31pd, L_31pv, L_d23ab, L_23d, R_d23ab, R_v23ab, R_23d, L_7m, R_7m), retrosplenial cortex (R_RSC), and inferior parietal lobe (L_PGs, R_PGs, R_PGi). Similarly, the structural analysis of the DMN identified a significant latent variable responsible for 29 % of the variance, with key ROIs including the medial prefrontal cortex (L_10r, L_p32, L_s32, R_10r, R_s32), posterior cingulate cortex (L_23d, L_31pv, R_d23ab, R_v23ab, R_31pv), lateral temporal cortex, and inferior parietal lobe (L_PFm, R_PFm, L_PGi, R_PGi, L_IP1, R_IP1). The relationships between each latent variable and their respective correlations with mFNAME performance are depicted in Figure 3.

## 4 Discussion

Alongside the surge in popularity of the FNAME task in cognitive aging research, our study advances the understanding of the task's psychometric properties in young adults and the neural correlates of task performance in cognitively impaired older adults. Critically, findings from Experiment 1 demonstrate high alternate form reliability of eight newly developed parallel versions of the mFNAME for all outcome measures (ICC = 0.8 to 0.9). Additionally, we report an absence of significant practice effects from separate analyses conducted to examine changes in performance with increased task exposure. These findings underscore the suitability of mFNAME for longitudinal studies with strong potential to detect disease progression across the early stages of the AD continuum and monitor treatment effects.

Distinct from this interrogation of alternate form reliability and practice effects, Experiment 2 yielded novel findings that advance our understanding of how functional and structural connectivity profiles correspond with associative memory in cognitively impaired older adults.

In the first step of this analysis, we employed graph theoretical analysis to identify which, if any, network(s) were significantly associated with mFNAME performance. This mathematical approach provides a useful lens to elucidate elements of the brain's structural and functional architecture that sustain complex cognitive processes. More specifically, this approach examines the brain's delicate balance between ensuring resilience against disruptions (local efficiency), facilitating information integration across varied regions (global efficiency), and preserving distinct modules to maximize specificity (system segregation) (Deery et al., 2023). Of the networks evaluated, this preliminary analysis revealed that only the network organization of the DMN and MTN was significantly associated with mFNAME performance. When examining structural connectivity profiles, we observed that global efficiency and local efficiency metrics for both the DMN and MTN significantly corresponded with mFNAME performance. Furthermore, unique to the functional topology of these networks, we observed that system segregation was significantly associated with face–name associative memory.

Together, these results reveal that both the structural and functional topology of the DMN and MTN are closely related to associative memory performance in MCI. These findings are congruent with reports from studies leveraging graph theory to survey the influence of network architecture on cognitive aging more broadly. Studies employing this methodology consistently report that age-related cognitive deficits are associated with less segregated, less modular, and less efficient network organization (see review by Deery et al., 2023). For example, higher system segregation of major functional networks reportedly confers cognitive resilience in both healthy older adults and patients with AD (Chan et al., 2014; Ewers et al., 2021). Our findings are consistent with this literature, as measures indicative of poorer system segregation in the DMN and MTN corresponded with worse performance on the mFNAME. These subtle changes in network organization revealed by graph theory may reflect *dedifferentiation (i.e., diffuse, nonspecific recruitment of brain regions)* and/or *compensation* (i.e., the need for additional neural resources to complete a task) (Deery et al., 2023). Higher global and local efficiency (i.e., more connections between nodes) reportedly confers resilience for information transfer and integration in the presence of disruptions (Achard and Bullmore, 2007), which also aligns with our findings. Such disruptions can occur as a natural consequence of age and multiply in pathological conditions such as AD. Supporting this notion, a recent investigation of preclinical AD reported that increased local efficiency in the DMN was associated with better memory performance in the presence of Aβ (Adams et al., 2023).

The identification of the DMN and MTN reflects well-established roles for these networks in the supporting memory throughout the aging continuum. The MTN fundamentally enables memory functions, comprising connectivity between the entorhinal, perirhinal, parahippocampal, and hippocampal cortices. The MTN dynamically balances the modularity and segregation necessary to facilitate its specialized functionality while remaining integrated with distributed brain systems through two specific networks converging on hippocampal structures: the anterior-temporal (AT) and the posterior–medial (PM) networks (de Flores et al., 2022; Deery et al., 2023). The AT connects the MTN with the temporopolar, orbitofrontal, and medial prefrontal cortices, while the PM provides connectivity to the precuneus, retrosplenial, and posterior cingulate cortices (Ranganath and Ritchey, 2012). This assimilation with distributed cortical structures provides a notable overlap between the MTN and DMN. Studies have reported that the MTN functions as a subsystem within the DMN, with the parahippocampal cortex serving as the hub that mediates its convergent connectivity (Ward et al., 2014). Although the DMN is principally characterized by its synchronous activation at “rest” that underlies internal mentation, this distributed network is increasingly recognized for its supportive role in memory functions (Raichle, 2015). The DMN's footprint provides connectivity between the posterior midline (precuneus/posterior cingulate), medial prefrontal, inferior parietal, and medial temporal cortices, facilitating the information integration necessary for encoding and retrieving associative memories (Smallwood et al., 2021). Furthermore, contextualizing our findings with the targeted application of the mFNAME task, both the DMN and MTN are noted for their vulnerability to the senescence that accompanies typical aging and AD pathology (Andrews-Hanna et al., 2007; Berron et al., 2020; Buckner et al., 2008; de Flores et al., 2022; Vlassenko et al., 2010). In summary, the MTN, crucial for episodic memory, supports binding discrete pieces of information, such as face–name pairs, into unified representations, a process central to tasks such as FNAME (Westerberg et al., 2012). Meanwhile, the DMN, known for its integration and abstraction capabilities, facilitates the extraction of commonalities across experiences, transforming specific associations into generalized knowledge. This dynamic interplay between the MTN's binding mechanisms and the DMN's integrative functions underscores how these networks collaboratively support the formation and contextualization of associative memories (Ward et al., 2015).

After identifying mFNAME-related networks, we employed partial least squares analysis (PLSC) to empirically characterize how variations in their functional and structural connectomes correspond with mFNAME performance. In the MTN, this yielded latent variables accounting for 44% and 31% of the covariance between associative memory scores and the functional and structural connectomes of this network, respectively. This was followed by a bootstrapping analysis to identify nodes that may act as critical hubs for associative memory. In the functional MTN, improved task performance was associated with enhanced connectivity between hippocampal subfields (CA3, GC_DG, CA4), the temporo-parietal-occipital junction (TPOJ3), the parietal lobe (7PL, PGp), and the posterior cingulate cortex (DVT, ProS, POS1, POS2) (Figure 2). The strength of white matter connectivity between a similar set of ROIs was associated with associative memory performance, with slightly denser clustering in subcortical structures of the MTL. Specifically, beyond connectivity to distributed cortical regions (i.e., PCV, POS2, ProS, 7PL, 7Pm, PGp), connections between the parahippocampus (PHA3), hippocampal subfields (CA3, GC_DG, CA1, CA4), the entorhinal cortex (EC), and perirhinal cortex (PeEc) in the MTL were also associated with the performance (Figure 2).

Relatedly, existing literature highlights the inferior longitudinal fasciculus (ILF) in facial recognition and memory (Burkhardt et al., 2023). The ILF serves as a major neural conduit linking memory structures in the MTL with distal brain regions, spanning from the anterior temporal pole to the posterior parietal and occipital regions (Sali et al., 2018). Our structural MTN PLSC analysis also coincides with the structural connectivity distribution of the ILF, linkage the bilateral PGp (posterior part of the inferior parietal lobe) to medial temporal subregions (PH3: parahippocampus, PeEC: perirhinal ectorhinal cortex). Notably, the ILF reportedly accounts for 40% of the long-range white matter pathways that pass through the hippocampus (Maller et al., 2019). Corresponding with this prominent hippocampal integration, disrupted connectivity along the ILF is commonly associated with memory deficits in older adults with and without AD (Kantarci et al., 2011; Kitamura et al., 2013; Luo et al., 2020; Madden et al., 2012; Sasson et al., 2013). Beyond its densely threaded connections to nodes in the MTN, the ILF's long projections bridge disparate brain regions that are a highly member for face–name binding (Sali et al., 2018). Anteriorly, it projects to the temporal pole, a region integral for linking faces with personal identity information (Tsukiura et al., 2010; Von Der Heide et al., 2013a). Posteriorly, the ILF projects to visual areas in the occipital cortex and adjacent structures such as TPOJ3, which is preferentially activated in response to viewing faces (Baker et al., 2018). The integral role of the ILF in face–name associative memories is supported by findings from our recent DTI-guided repetitive transcranial magnetic stimulation (rTMS) study in older adults, which stimulated superficial cortical tissue in the parietal lobe that demonstrated connectivity to the hippocampus via the ILF. With subject-specific stimulation sites, this rTMS protocol reportedly enhanced hippocampal functional connectivity and improved FNAME performance in participants with MCI (Chen et al., 2022).

With respect to the DMN, our sequential PLSC and bootstrapping analyses identified patterns of functional and structural connectivity that accounted for 37% and 29% of the covariance with mFNAME performance, respectively. Bootstrapping ratios with the highest values highlighted nodes in the prefrontal (p32, s32, 31pv, 47l, 47m, 10r), posterior cingulate (d23ab, v23ab, 23d), precuneus (7m), and parietal (PFm, PGi, POS1) cortex. Known for its extensive connectivity, the postero-ventral division of the cingulate emerges as a compelling nexus for the regions implicated by our analyses. As elucidated by Rolls et al., this neural hub facilitates the integration of memory processes in disparate cortical regions with those in hippocampal structures to facilitate the encoding and retrieval of new associations (Rolls et al., 2023). Notably, some of the highest bootstrapping ratios emerged for connectivity between structures in the prefrontal cortex and the posterior cingulate cortex (PCC), which complements the well-established involvement of the prefrontal cortex in associative memory (Becker et al., 2015; Guardia et al., 2023; Simons and Spiers, 2003). Indeed, multiple studies evaluating the regional contributions of age-related cortical atrophy to behavioral deficits reported that the gray matter volume in the prefrontal cortex, more so than the MTL, has the strongest correspondence with associative memory (Becker et al., 2015; Guardia et al., 2023). Additional work has highlighted the left superior frontal gyrus (L_p32), specifically, as a convergence region for binding verbal and non-verbal material like that of the FNAME task (Klamer et al., 2017). Relatedly, the angular gyrus (PGs, PFm, PGi) is also known to be particularly involved in binding cross-modal episodic features and representing high-level face information (Lee and Kuhl, 2016; Tibon et al., 2019). Lastly, our results emphasize orbitofrontal regions (areas 47 and 10), which are structurally connected to the temporal cortex via the uncinate fasciculus (UF). This finding aligns with the purported functionality of the UF for “temporal lobe-based mnemonic associations (e.g., an individual's name + face + voice)” (Von Der Heide et al., 2013b) and previous reports implicating the structural integrity of the UF in face–naming abilities (Metoki et al., 2017; Papagno et al., 2011).

By leveraging the graph-based properties of DMN and MTN organization, these networks emerge as promising targets for interventions such as repetitive transcranial magnetic stimulation (rTMS) or transcranial direct current stimulation (tDCS) in this population. Prior research has demonstrated the efficacy of non-invasive brain stimulation (NIBS) in modulating network-level activity to improve memory performance, particularly in aging and clinical populations with memory impairments (Chen et al., 2022; Tambini et al., 2018; Wang et al., 2014). Future research could incorporate graph theory analyses to enhance the application of NIBS therapies in this population and increase our understanding of the intervention's effects. For example, a recent study in patients with clinical depression reported that network properties identified through graph theory were predictive of rTMS treatment response (Klooster et al., 2019). Other studies in various clinical populations indicate that graph-based properties of network organization can be “renormalized” following NIBS therapies. For example, in correlation with improved treatment response, inefficient small-world properties and aberrant functional segregation observed in insomnia patients at baseline were restored following rTMS to resemble the network organization of healthy controls (Qi et al., 2022). Similar findings exist in the clinical depression literature, where rTMS treatment response is associated with renormalization in network topology discerned by graph-based properties (Zhang et al., 2025). To our knowledge, graph theory analyses have yet to be integrated into studies applying NIBS in populations with age-related cognitive impairment. Though previous studies have reported that individualized rTMS can improve FNAME performance and modulate functional connectivity in patients with MCI (Chen et al., 2022), future work is needed to determine whether these behavioral improvements are associated with shifts in global efficiency, local efficiency, and/or system segregation within the networks identified by the present work.

Various caveats should be taken into account when interpreting our results. First, the alternate form reliability of the eight newly developed parallel versions of mFNAME (Experiment 1) was only assessed in younger adults due to the enhanced feasibility afforded by this population. Our cohort of cognitively impaired older adults in Experiment 2 completed a single mFNAME assessment, utilizing a randomly selected version of the task. Second, the absence of disease-specific biomarkers in Experiment 2 limits the interpretability of our findings. It is unclear, for example, how the connectomic correlates of mFNAME performance identified in the present work may be influenced by disease-specific neuropathology.

## 5 Conclusion

The high alternate form reliability of the eight parallel versions of mFNAME reported in Experiment 1 underscores the task's potential as a valuable tool for early detection and monitoring of cognitive decline along the continuum of AD and related dementias. Experiment 2 provides novel results, yielding insights into how the connectivity of the MTN and DMN is associated with mFNAME task performance. Graph theoretical analysis revealed that global and local efficiency metrics for each network's structural connectome were significantly associated with task performance, while system segregation emerged as the only significant correlate of mFNAME for each network's functional connectome. Subsequent PLSC and bootstrapping analyses revealed more granular connectomic features within each network that was associated with task performance. Future work is needed to (1) evaluate mFNAME reliability in aged populations, and (2) integrate disease-specific biomarkers to ascertain potential interactions between neuropathology and the connectomic correlates of mFNAME identified in Experiment 2.

## Data Availability

The original contributions presented in the study are included in the article/Supplementary material, further inquiries can be directed to the corresponding author.
