# Supplementary material for: Advancing the modified face name associative memory exam in cognitive aging research: insights into connectomic correlates and task reliability
Source: Front Aging Neurosci. 2025 Jul 28;17:1592678. doi: 10.3389/fnagi.2025.1592678 (PMC12336139; doi:10.3389/fnagi.2025.1592678)
Supplement: Supplementary file 1 [file Data_Sheet_1.docx]

**Supplementary Materials**

Supplementary Table 1. Outcomes of regression analyses examining the relationships between structural network properties (global efficiency, local efficiency, and system segregation) and mFNAME performance metrics, specifically accuracy and sensitivity index D1.

| Structural Network | | Accuracy | | D1 | |
| --- | --- | --- | --- | --- | --- |
|  |  | R-value | p-value | R-value | p-value |
| Global Efficiency | Visual Network | 0.2816 | 0.1248 | 0.2639 | 0.1513 |
|  | Somatosensory Motor network | 0.1733 | 0.3511 | 0.1943 | 0.2947 |
|  | Salient network | 0.2827 | 0.1234 | 0.1449 | 0.4367 |
|  | Language network | 0.2862 | 0.1185 | 0.1941 | 0.2954 |
|  | Frontal Parietal Network | 0.3350 | 0.0654 | 0.1847 | 0.3199 |
|  | Auditory Network | 0.7267 | 0.5081 | 0.0191 | 0.9187 |
|  | Medial temporal network | 0.5416 | 0.0152 * | 0.5663 | 0.0239 * |
|  | Default mode network | 0.6012 | 0.0075 ** | 0.5059 | 0.0376 * |
| Structural Network | | Accuracy | | D1 | |
|  |  | R-value | p-value | R-value | p-value |
| Local Efficiency | Visual Network | 0.3110 | 0.7083 | 0.3075 | 0.1924 |
|  | Somatosensory Motor network | 0.1442 | 0.4389 | 0.1540 | 0.4080 |
|  | Salient network | 0.3010 | 0.7988 | 0.1817 | 0.3279 |
|  | Language network | 0.3551 | 0.3995 | 0.3279 | 0.1456 |
|  | Frontal Parietal Network | 0.4104 | 0.1747 | 0.2275 | 0.2184 |
|  | Auditory Network | 0.0655 | 0.1 | 0.0001 | 0.1 |
|  | Medial temporal network | 0.4886 | 0.0239 * | 0.3421 | 0.0871 |
|  | Default mode network | 0.5274 | 0.0108 * | 0.4756 | 0.0376 * |
| Structural Network | | Accuracy | | D1 | |
|  |  | R-value | p-value | R-value | p-value |
| System Segregation | Visual Network | -0.0691 | 0.7213 | -0.0910 | 0.6386 |
|  | Somatosensory Motor network | 0.0822 | 0.6713 | -0.0166 | 0.9318 |
|  | Salient network | 0.0861 | 0.6569 | -0.0776 | 0.6891 |
|  | Language network | 0.1741 | 0.3664 | 0.0571 | 0.7685 |
|  | Frontal Parietal Network | 0.18409 | 0.3391 | 0.1289 | 0.5051 |
|  | Auditory Network | 0.0164 | 0.9327 | -0.2309 | 0.2281 |
|  | Medial temporal network | 0.2980 | 0.1672 | 0.3631 | 0.0884 |
|  | Default mode network | 0.0609 | 0.7675 | -0.0602 | 0.7699 |

Supplementary Table 2. Outcomes of regression analyses examining the relationships between functional network properties (global efficiency, local efficiency, and system segregation) and mFNAME performance metrics, specifically accuracy and sensitivity index D1.

| Functional Network | | Accuracy | | D1 | |
| --- | --- | --- | --- | --- | --- |
|  |  | R-value | p-value | R-value | p-value |
| Global Efficiency | Visual Network | 0.3762 | 0.1223 | 0.2389 | 0.2121 |
|  | Somatosensory Motor network | 0.2846 | 0.1541 | 0.2243 | 0.2420 |
|  | Salient network | 0.3519 | 0.1223 | 0.2803 | 0.1408 |
|  | Language network | 0.3578 | 0.1222 | 0.1845 | 0.3377 |
|  | Frontal Parietal Network | 0.3608 | 0.1223 | 0.2016 | 0.2941 |
|  | Auditory Network | 0.2798 | 0.1541 | 0.1723 | 0.3715 |
|  | Medial temporal network | 0.0322 | 0.8587 | -0.1469 | 0.4145 |
|  | Default mode network | 0.2124 | 0.4705 | 0.0525 | 0.7713 |
| Functional Network | | Accuracy | | D1 | |
|  |  | R-value | p-value | R-value | p-value |
| Local Efficiency | Visual Network | 0.4154 | 0.1999 | 0.3214 | 0.3422 |
|  | Somatosensory Motor network | 0.3331 | 0.6189 | 0.1956 | 0.3090 |
|  | Salient network | 0.2900 | 0.6032 | 0.1449 | 0.4531 |
|  | Language network | 0.3627 | 0.4249 | 0.2133 | 0.2664 |
|  | Frontal Parietal Network | 0.8729 | 0.3473 | 0.1465 | 0.4481 |
|  | Auditory Network | 0.5187 | 0.3362 | 0.2179 | 0.2460 |
|  | Medial temporal network | 0.1910 | 0.2949 | 0.0184 | 0.9200 |
|  | Default mode network | 0.1517 | 0.4071 | -0.0306 | 0.8677 |
| Functional Network | | Accuracy | | D1 | |
|  |  | R-value | p-value | R-value | p-value |
| System Segregation | Visual Network | -0.1559 | 0.4471 | -0.1579 | 0.4409 |
|  | Somatosensory Motor network | 0.0088 | 0.9661 | -0.1385 | 0.4997 |
|  | Salient network | -0.1772 | 0.3865 | -0.1845 | 0.3369 |
|  | Language network | -0.2298 | 0.2587 | -0.1449 | 0.4801 |
|  | Frontal Parietal Network | -0.3095 | 0.1238 | -0.1834 | 0.3678 |
|  | Auditory Network | -0.1237 | 0.5468 | -0.1731 | 0.3978 |
|  | Medial temporal network | 0.5644 | 0.0040** | 0.5482 | 0.0055 ** |
|  | Default mode network | 0.4103 | 0.0464 * | 0.3434 | 0.1003 |
